# First Report on Isolation and Characterization of *Leishmania major* from *Meriones hurrianae* (Rodentia: Gerbillidae) of A Rural Cutaneous leishmaniasis Focus in South-Eastern Iran

**DOI:** 10.5812/ircmj.6974

**Published:** 2013-09-05

**Authors:** Hamid Kassiri, Saied Reza Naddaf, Ezat–Aldin Javadian, Mehdi Mohebali

**Affiliations:** 1 Department of Medical Entomology and Vector Control, School of Health, Ahvaz Jundishapur University of Medical Sciences, Ahvaz, IR Iran; 2 Department of Parasitology, Pasteur Institute of Iran, Tehran, IR Iran; 3 Department of Medical Entomology and Vector Control, School of Public Health, Tehran University of Medical Sciences, Tehran, IR Iran; 4 Department of Medical Parasitology, School of Public Health, Tehran University of Medical Sciences, Tehran, IR Iran

**Keywords:** *Meriones hurrianae*, *Leishmania major*, Molecular Characterization, RAPD-PCR, Reservoir Host, Iran

## Abstract

**Background:**

Zoonotic Cutaneous Leishmaniasis (ZCL) is an endemic health problem in many rural areas of Iran, with doubled number of incidences over the last decade. Different species of rodents serve as natural reservoir host for ZCL. The disease is considered as a major health problem in rural areas of Mirjaveh, Chabahar, and Konarak Counties of Sistan va Baluchistan Province.

**Objectives:**

This study describes the identity of *Leishmania* species, isolated from *Meriones hurrianae* from Chabahar County using RAPD-PCR methodology.

**Materials and Methods:**

Rodents were entrapped by live traps baited with roasted walnut, tomato, and cucumber during spring and summer. All rodents were identified based on external features including fur color, ears characteristics, tail length, hind feet, body measurements, and internal features of teeth and cranium. Giemsa-stained impressions from rodents’ ears were examined for amastigotes microscopically. The samples from infected rodents were cultured in NNN+LIT medium and then the harvested parasites at the stationary phase were subjected to DNA extraction followed by amplification with RAPD-PCR.

**Results:**

All the 28 entrapped animals were identified as *M. hurrianae*. Five animals showed to harbor Leishmania parasite by microscopy. Leishmania DNA isolated from five *M. hurrianae* produced distinctive bands of * L. major* with four primers. However, the products that were amplified with primers AB1-07, 327, and 329 were stable and reproducible. This is the first report on the isolation and identification of *L. major* from *M. hurrianae* from Iran.

**Conclusions:**

Regarding infection rate of 17.8%, *M. hurrianae* seems to play the major role in the maintenance and transmission of disease to humans in this area.

## 1. Background

Leishmaniases, most zoonotic, are complex worldwide diseases caused by more than 20 species of *Leishmania* belonging to the family Trypanosomatidae (order Kinetoplastida). *Leishmania* parasites are transmitted via the infective bites of about 30 different species of sand flies (subfamily Phlebotominae) (1, 2).

Leishmaniasis is prevalent in many tropical and sub-tropical areas covering about 88 countries with approximately 350 million people at risk of acquiring the infection (3). There are four main forms of leishmaniasis: Visceral Leishmaniasis (VL), Mucocutaneous Leishmaniasis (MCL), Diffuse Cutaneous Leishmaniasis (DCL), and Cutaneous Leishmaniasis (CL) (3). CL is the most common form of disease; Patient generally presents with one or several ulcer (s) or nodule (s) in the skin. Over 90% of cases of CL have been reported in Afghanistan, Iran, Iraq, Saudi Arabia, Syria, Algeria, Brazil, and Peru (4).

Three species of *Leishmania* parasites are etiological agents of CL in the old world: *L. major*, *L. tropica* and *L. aethiopica* (5). *L. major* and *L. tropica* are the causing agents for Zoonotic Cutaneous Leishmaniasis (ZCL) and Anthroponotic Cutaneous Leishmaniasis (ACL) are prevalent in Iran with infection prevalence ranging from 1.8% to 37.9% in different Provinces (6).

ZCL is endemic in many rural districts of Iran affecting 17 out of 31 Provinces. Various species of rodents (family: Gerbillidae) have been incriminated as natural reservoir hosts of ZCL in Iran. *Rhombomys opimus* is known as the primary reservoir in central, north, and north eastern Iran (7, 8). *Meriones libycus* plays the secondary role as reservoir host alongside *R. opimus* in central Iran (9) and is also known as primary host in Arsanjan, Neiriz, Marvdasht counties (Fars Province), Ardestan County (Isfahan Province) and Qom County (Qom Province) (10-13). In Natanz County (Isfahan Province), besides the two above mentioned species, *Leishmania* infection has been detected in *Meriones persicus*. In Damghan area (Semnan Province), north Iran, *Nesokia indica*, *M. libycus*, and *R. opimus* are known as main reservoir hosts (14, 15). In south, west, and south western Iran *Tatera indica* is the primary host along with *N. indica* and *M. libycus* as the secondary hosts (16, 17). In southeastern Iran *M. hurrianae* and *T. indica* are primary and secondary reservoir hosts for ZCL, respectively (18). There are also records for infection of *Gerbillus ssp.* and *Rattus norvegicus* to *L. major* in Fars Province (19, 20), and *Gerbillus nanus* in Jask County (Hormozgan Province) (21). One of the major problems for control of ZCL is lack of knowledge about the nature of *Leishmania* parasites in reservoir hosts population.

## 2. Objectives

This study describes isolation of *Leishmania *parasite from *M. hurrianae* in rural areas of Chabahar County, Sistan va Baluchistan Province followed by identification of the species using DNA analysis.

## 3. Materials and Methods

### 3.1. Study Area

Chabahar County is located on the shore of Oman Sea littoral in southeastern province of Sistan va Baluchistan, Iran. This County is a low landing area; with geographical coordinates of 25° 17’ North, 60° 38’ East. The climate of this area is classified as very warm desert due to its low annual precipitation. The average annual temperature and humidity are 36.4ºC and 75.9%, respectively. Chabahar County covers an area of 24,729 Km^2^, with 230,000 residents. The majority of the county’s inhabitants are ethnic Baluch, speaking the Baluchi language. This descriptive cross–sectional study was carried out in 1997 in 3 villages (Negor , Pollan and Noubandian) of Chabahar County, Dashtiyari Division of Chabahar, where CL emerged as an endemic disease.

### 3.2. Sample Collection

Three trained persons with the same educational and professional levels were hired for rodents catching all over the study period. The ethical principles of this research were investigated and discussed in research committee of medical entomology department and necessary modifications made, faced to be approved. The sample size was selected at the minimum accepted levels because of the ethical aspects related to the animal rights. Before killing the rodents for reducing their painful feeling, we used Ether or Chloroforme as an anesthetized agent. The sample size was chosen using below Formula:

$$n=\frac{{\text{1.96}}^{2}\times \text{0.05}\left(\text{0.95}\right)}{\text{0.0065}}=\text{28}$$

Also, expected power was calculated 75%. Based on our research design all sampling were done using simple randomized approach. SPSS 16 and MINITAB 14 softwares were applied for statistical analysis of data.

Rodents were entrapped by live traps baited with roasted walnut, tomato, and cucumber during spring and summer. The traps were placed at sunset and collected at dawn. All rodents were identified based on external features including fur color, ears characteristics, tail length, hind feet, body measurements, and internal features of teeth and cranium.

In this research, infection of the rodents observed through microscopic and PCR (Polymerase Chain Reaction) method. Impression smears were taken from rodent’s ears, stained with Giemsa, and examined microscopically for amastigotes. The samples from infected rodents were cultured in Novy–MacNeal–Nicolle (NNN) medium, liver infusion broth tryptose (LIT) and then checked twice a week for promastigotes. The positive cultures were then transferred to RPMI 1640 medium supplemented with 10% heat inactivated fetal calf serum. The parasites were harvested at the stationary phase and kept at -20ºC until used.

### 3.3. DNA Extraction

The harvested parasites were washed in cold sterile PBS (pH 7.2) several times. The recovered pellet was re-suspended in 300 μL cell lysis buffer (50 mM NaCl, 50 mM EDTA, 1% SDS, and 50 mM Tris-HCl, pH 8.0) with 20 μL of 20 mg/mL proteinase K and incubated at 55ºC overnight. DNA was extracted from lysate with phenol/chloroform followed by ethanol precipitation. The DNA was re-suspended in distilled water and working solutions were adjusted to 5 ng/μL in distilled water.

### 3.4. RAPD-PCR Analysis

The RARD-PCR assays were performed as outlined by others (14 , 22). Each 25 μL reaction contained 10 mM Tris-HCl, pH 8.3, 50 mM KCl_2 _, 2 mM MgCl_2 _, 200 μM of each dNTP, 50 pmol of one of the primers (Table 1), 1 unit of Taq DNA polymerase, and 10 ng of DNA. Reactions were overlaid with 25 μL of mineral oil and amplified with a thermocycler programed for one cycle at 94°C for 5 min followed by 45 cycles of denaturation at 94°C for 1 min, annealing at 37°C to 38°C for 1 min, and extension at 72°C for 2 min and a final extension step at 72°C for 5 min. A negative control, containing all components except DNA, was included in all assays. Amounts of 8-10 µl of amplicons were run alongside a DNA size marker (Roche, Germany) on a Electrophoresis (1.2% agarose gel containing ethidium bromide) and visualized on a UV Transilluminator. Resulting bands were examined and photographed. 

**Table 1.** The Primers Used in RAPD-PCR Analysis

No.	Code	Sequence	% GC
**1**	AB1-07	GGT GAC GCA G	70
**2**	327	ATA CGG CGT C	60
**3**	329	GCG AAC CTC C	70
**4**	335	TGG ACC ACC C	70

## 4. Resutls

**Figure 1.**
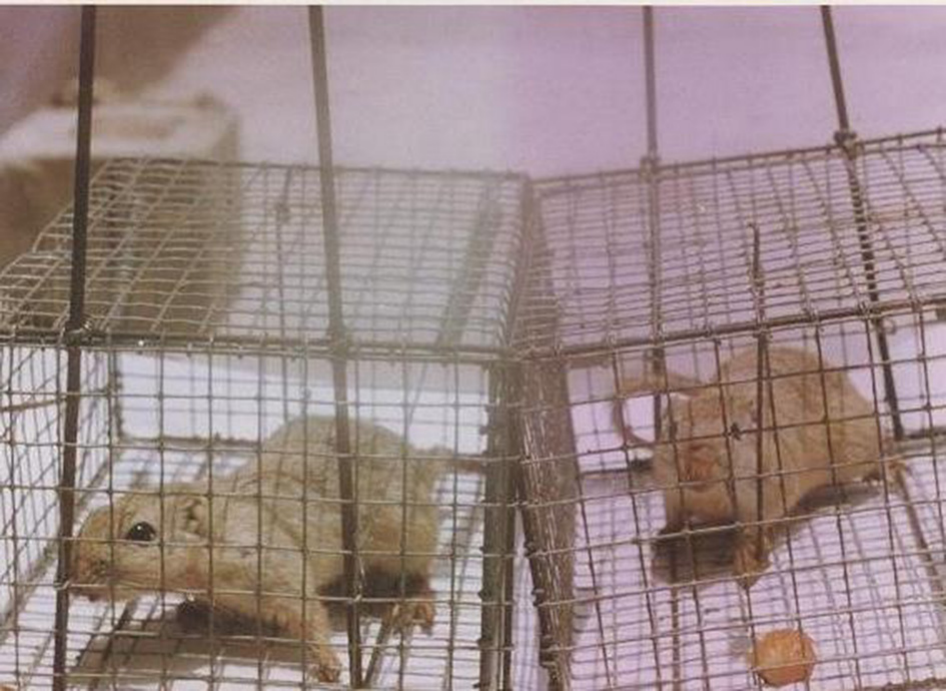
Meriones hurrianae Entrapped in Chabahar County, Sistan va Baluchistan Province, Iran

**Figure 2.**
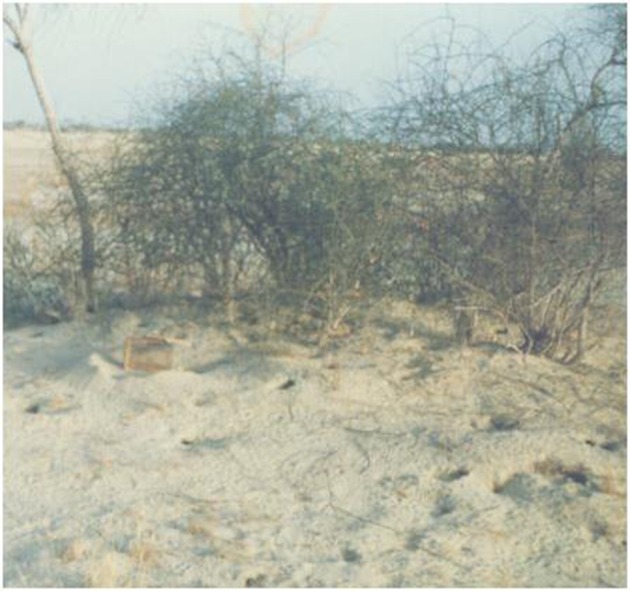
Typical Rodent Burrows of Meriones hurrianae, Chabahar County, Sistan va Baluchistan Province, Iran

**Figure 3.**
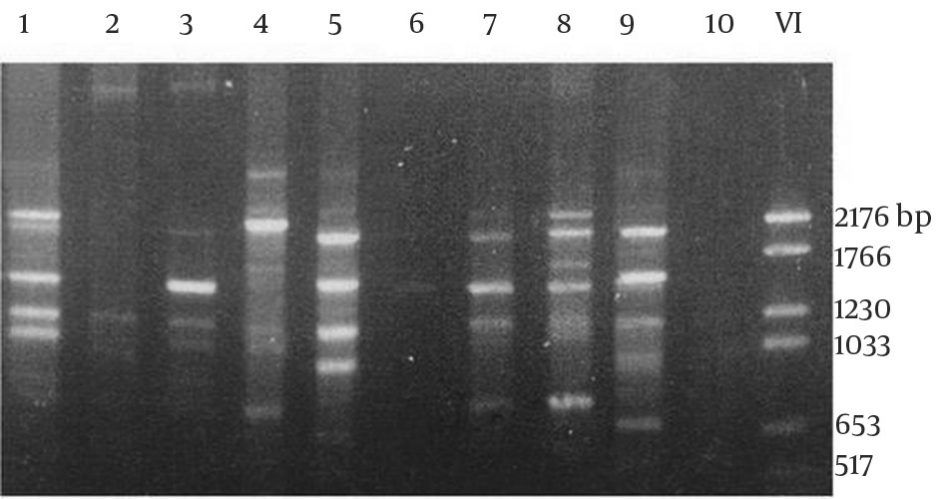
Electrophoresis of Gene Fragments Amplified With RAPD-PCR Using the Primer 329 Lanes 1, 4, 6, and 8 different strains of *L. major*; lane 2 *L. donovani*; 5 and 9 different strains of *L. tropica*; lane 3, the strain originated from *Mer.hurrianae* (This study); lane10, control; lane 11, DNA size marker VI (Roche, Germany)

**Figure 4.**
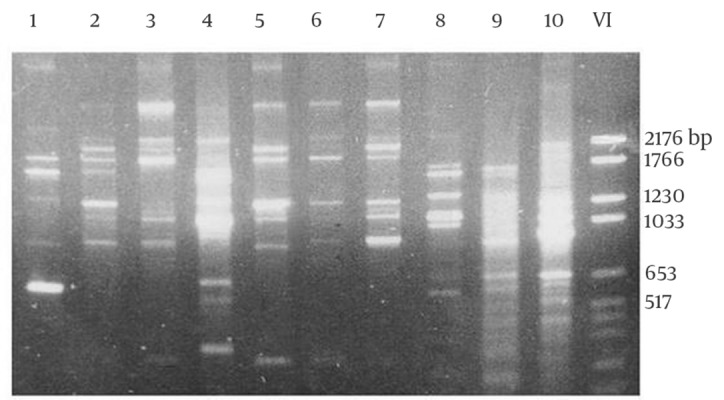
Electrophoresis of Gene Fragments Amplified with RAPD-PCR Using the Primer 327 Lanes 1, *L. infantum*; Lanes 2, 5, 6 and 7 different strains of *L. major*; lanes 4, 8,9, and 10 different strains of *L. tropica*; lane 3, the strain originated from *M. hurrianae* (This study); lane 11, DNA size marker VI (Roche, Germany)

**Figure 5.**
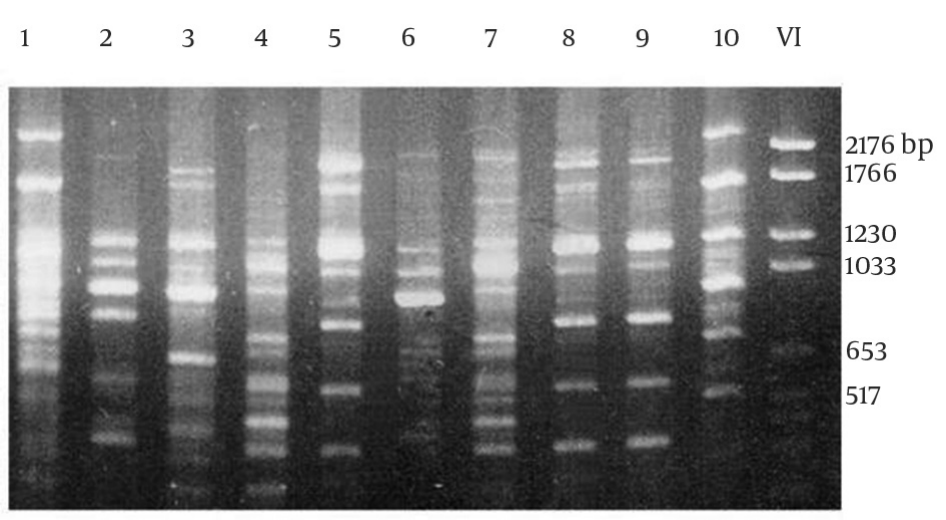
Electrophoresis of Gene Fragments Amplified with RAPD-PCR Using the Primer AB1-o7 Lanes 1 and 10 *L. infantum*; Lanes 2, 3, and 6 different strains of *L. major*; lanes 4, 7, 8, and 9 different strains of L. tropica; lane 5, the strain originated from *M. hurrianae* (This study); lane 11, DNA size marker VI (Roche, Germany)

A total of 28 rodents were collected with live traps from three villages. All the animals were identified as *M. hurrianae *(Figure 1 and Figure 2). Microscopical examination of Giemsa- stained impression smears from rodents ears showed that five out of 28 animals were infected with amastigotes. The parasites from 5 animals were grown successfully in culture medium. DNA amplification with RAPD-PCR of DNA from 5 isolates yielded distinctive bands that were characteristic of *L. major* with four primers, but the products that were amplified with primers AB1-07, 327, and 329 were stable and reproducible in all assays (Figure 3, Figure 4 and Figure 5). This is the first report on the isolation and identification of *L. major* from this rodent species in Iran. 

## 5. Discussion

WHO (World Health Organizaton) introduces the leishmaniasis as one of the six important worldwide infectious diseases. So, doing researches on leishmaniasis and focusing on its several aspects is highly recommended. Movement of immune people to endemic regions, presence of people with lesion, increasing population, existence of rodent burrows and presence of infected sand flies have made the most suitable conditions for disease transmission, in the Chabahar County.

*Leishmania major*, the etiological agent of ZCL, is the disease of rodents in arid and savannahs from the Old World. In Iran, different species of rodents belonging to family Gerbillidae have been incriminated as reservoir hosts of this parasite and new species are increasingly added to the list. Zoonotic Cutaneous leishmaniasis has also been reported during recent decades in rural areas of Chabahar County, Sistan va Baluchistan Province, southeast Iran, bordering with Pakistan.

The most prevalent sand fly species in this area, *Phlebotomus papatasi* and *P. salehi*, were found to be infected with *L. major* with RAPD-PCR assay (23). The most prevalent rodent species in the area, *M. hurrianae* , has already been identified as a reservoir host of *L. major* in India (5). This species has been recorded in the Thar Desert of India, Iran, and Pakistan. In Pakistan it has been seen in Punjab, Sindh, Baluchistan and north west frontier Province. It is questionably present in Afghanistan at Kelat-i-Ghilzai between Ghazni and Kandahar (24).

In this study, *Leishmania* parasites were isolated from *M. hurrianae* in Chabahar County and were identified as *L. major* with RAPD-PCR assey. Regarding infection rate of about 17.8% in *M. hurrianae*, this species can be considered as the main reservoir host of ZCL and the principle source of human infection in this area. With concern to the results of this research , it is obvious that the ZCL foci in Chabahar County exist and this form is dominant. As *L. major* (dominant species), is a zoonotic parasite, then we should have special concern related to the rural areas immigrants and also rodent control in these regions. By this respect, the PCR molecular technique is a highly reliable procedure for diagnosis of *Leishmania *specie. In PCR method, as DNA of *Leishmania *species is examined, therefore its sensitivity and specificity are so high.
